# The metabolic advantage of being young and male in obesity treatment outcomes in mice

**DOI:** 10.1038/s44324-025-00065-2

**Published:** 2025-08-01

**Authors:** Amanda S. Dirnberger, Elen Yanina Aguirre-Rodriguez, Elias Carlos Aguirre-Rodriguez, John O. Degraft Hanson, Yanping Sun, Dave Delima, Benjamin F. Bykov, Aneirson Francisco da Silva, Marko Kraljević, Fernando Augusto Silva Marins, Ana BF Emiliano

**Affiliations:** 1 Department of Visceral Surgery, Clarunis, University Digestive Health Care Center Basel, St. Claraspital and University Hospital Basel, Basel, Switzerland; 2 School of Engineering and Sciences - UNESP - São Paulo State University, São Paulo, Brazil; 3 Morehouse School of Medicine, Atlanta, Georgia USA; 4 Oncology Precision Therapeutics and Imaging Core, Columbia University Herbert Irving Comprehensive Cancer Center, New York City, NY USA; 5 The Boys’ Latin School of Maryland, Baltimore, Maryland USA; 6 Department of Medicine, Columbia University Irving Medical Center, New York City, NY USA

**Keywords:** Diabetes, Obesity

## Abstract

Although diversity in clinical trials is important to test the efficacy of a treatment, weight loss trials rarely account for age and sex. To highlight this deficiency, we set out to test whether age and sex affect WAT mobilization after weight loss surgery or intermittent fasting, in an obese mouse model. Here we show that male sex, youth, and WAT transcriptomic plasticity are characteristics associated with improved weight loss outcomes. Conversely, aging impairs WAT mobilization and transcriptomic plasticity. Greater surgical weight loss is associated with changes in the expression of genes relevant to the IL17 inflammatory signaling pathway, angiotensin converting enzyme 2 (ACE2) signaling, lipolysis, carbohydrate metabolism and adipocyte differentiation. In conclusion, female sex and older age appear to hinder molecular processes necessary for the reversal of WAT expansion. Future studies should examine the relevance of these findings to human obesity therapeutics.

## Introduction

Despite the effectiveness of glucagon-like peptide-1 receptor agonists (GLP-1-RA) for weight loss, the obesity epidemic still is a major public health problem in the US. Low affordability and frequent treatment discontinuation limit the reach of GLP1RAs^1,2^. For many patients, weight loss surgery and caloric restriction continue to be realistic options. However, weight loss surgery’s effectiveness and durability can be quite variable^3^. Moreover, less than 5% of patients that lose significant weight on caloric restriction are able to maintain the weight loss^4,5^. Accordingly, weight loss surgery or caloric restriction should be recommended for patients that have a reasonable chance of responding favorably. It is not known whether baseline characteristics, such as age and sex, predict clinical outcomes.

Metabolism is highly influenced by sexual dimorphism. Men have a higher prevalence of diabetes than women^6^ and in most animal models used in biomedical research, males have a higher propensity for obesity and insulin resistance, including in the diet-induced obesity (DIO) mouse model used in this study^7^. Although obesity and insulin resistance are more prevalent in men, women with obesity appear to be more resistant to weight loss^8^. Men lose significantly more weight than women when placed on a low-calorie diet, achieving better cardiometabolic markers than women of the same age^9,10^. Much less is known about the effects of sex differences on weight loss surgery, as most trials examining weight loss surgery are not designed to detect sex differences^11–14^.

Aging is accompanied by body weight gain and fat mass accumulation in both humans and rodents^15^. Older people have a higher risk of becoming overweight and obese, a phenomenon that is tied to sarcopenia, which is the loss of muscle mass^16,17^. Sarcopenia is considered one of the main reasons older human adults with obesity have more difficulty losing weight^17^. The process of aging is characterized by increasing levels of systemic inflammation, higher circulating levels of pro-inflammatory cytokines, including tumor necrosis factor alpha (TNF-α), interleukin 6 (IL6) and interleukin 17 (IL17), similarly to obesity itself^18^. Weight loss surgery studies have conclusively shown that older patients experience more limited weight loss and a lower rate of diabetes remission than younger patients^19–21^.

The present study tested the hypothesis that sex and age differences in white adipose tissue (WAT) biology drive metabolic outcomes from weight loss therapies. Owing to an excellent safety profile and a high-resolution rate of obesity and its co-morbidities compared to diet and exercise, sleeve gastrectomy (SG) continues to be the most commonly performed bariatric procedure worldwide (55.4%)^22,23^. Intermittent fasting (IF) – which includes caloric restriction - can be an effective weight loss intervention and is routinely prescribed by medical providers^24^. To test our hypothesis, we measured the impact of sex and age on total percent weight loss, blood glucose change, energy intake, energy expenditure, locomotor activity, WAT depot re-distribution/loss, and WAT transcriptomic changes after SG and IF in diet-induced obese (DIO) mice. The control group consisted of mice that underwent sham surgery and were kept on an ad libitum diet, where the mice are allowed to eat at will.

## Results

### The impact of sex and age on weight loss

**Fig. 1:**
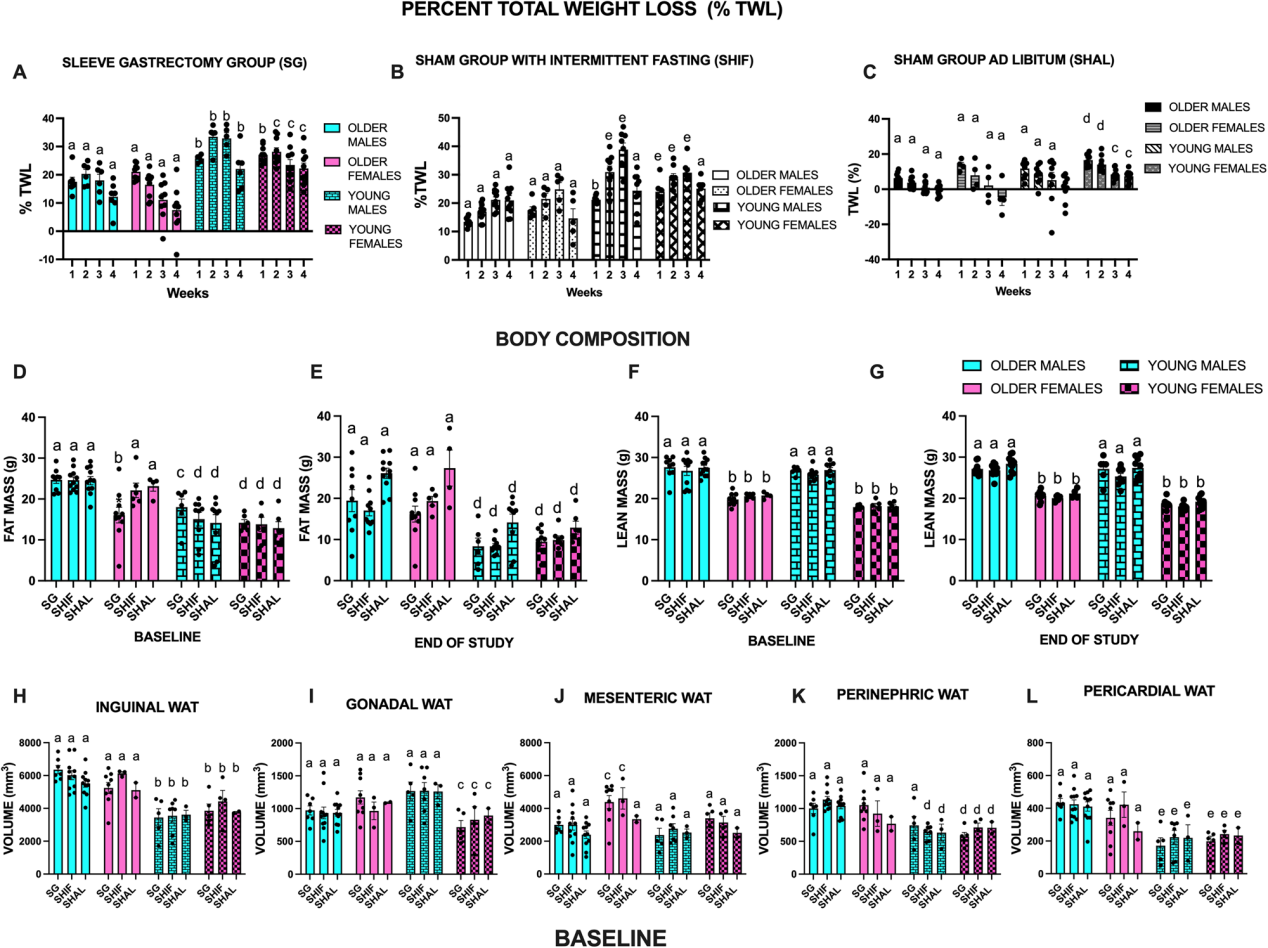
Weight Loss and Body Composition before and after SG, SHIF and SHAL. Two-Way ANOVA with Tukey’s tests for multiple comparisons (pairwise analysis). A - C. Percent total weight loss from baseline. **A** After SG, older males and females substantially differed from young males and young females in week 1 to week 4 (a versus b, *p* < 0.01, a versus c, *p* < 0.05). **B** In the SHIF group, older males and females differed from young males and females from weeks 1–3 (a versus b, *p* < 0.01; a versus e, *p* < 0.0001). **C** In the SHAL group, young females lost significantly more weight than all the other groups (a versus d, p < 0.001; a versus c, p < 0.05). **D, E** Fat Mass at baseline and end of study. Older mice had the highest fat mass at baseline and at the end of the study(a versus b, *p* < 0.001; a versus c, *p* < 0.05; a versus d, *p* < 0.0001). **F, G** Lean Mass at baseline and end of study. Males had higher lean mass at baseline and at the end of the study (a versus b, *p* < 0.0001). **H–L** Baseline iWAT, gWAT, mWAT, rWAT and cWAT volumes. Older mice had higher iWAT, rWAT and cWAT than younger mice (a versus b, *p* < 0.0001; a versus c, *p* < 0.05; a versus d, *p* < 0.01, a versus e, *p* < 0.001). *N* = 6-14 per group. Data expressed as means ± SEM. SG sleeve gastrectomy, SHIF sham surgery with intermittent fasting, SHAL sham surgery with ad libitum feeding. iWAT inguinal white adipose tissue, gWAT gonadal white adipose tissue, mWAT mesenteric white adipose tissue, rWAT perinephric white adipose tissue, and cWAT pericardial white adipose tissue.

One fundamental goal of our study was to quantify the influence of age and sex on weight loss after SG or sham surgery with intermittent fasting (SHIF). Younger SG or SHIF mice, of both sexes, achieved greater total percent weight loss than their older (middle-aged) counterparts (Fig. 1A, B) (please see Methods for the specific age in weeks at randomization). Total percent weight loss for younger mice was almost 10% higher than for older (middle-aged) mice after SG or SHIF. Unsurprisingly, there was only a small weight loss in the sham surgery with ad libitum feeding (SHAL) cohort. SHAL older (middle-aged) males had the lowest post-surgical weight loss, while SHAL young females lost the most weight, albeit at a much lower rate than SG or SHIF mice (Fig. 1C). Although sex was not preponderant as age in driving weight loss, males in the older (middle-aged) and younger SG groups trended towards higher weight loss than their female counterparts, especially on the second and third post-operative weeks (Fig. 1A, B). The influence of sex on weight loss was not as discernible in the SHIF cohort as in the SG group (Fig. 1B).

We also found that total percent weight loss correlated with total fat mass loss (Figs. 1D, E, 3M). Younger mice lost more weight and more fat mass than older mice. Lean mass remained essentially constant throughout the study for all groups (Fig. 1D–G). It is important to note that aging causes mice to gain fat free mass, unlike humans^15^. This fact may have offset more significant lean mass loss that would have otherwise happened. Both sex and age appeared to influence fat mass loss, as younger male mice that underwent SG or SHIF lost the most fat mass (Fig. 3M).

In addition, we measured the volume of specific WAT depots at baseline (Fig. 1H–L) and at the end of the study, 4 weeks later (Fig. 3A–R) with the use of a Bruker Tesla 9.4 preclinical MRI (in methods). The WAT depots measured included inguinal, gonadal, mesenteric, perinephric and pericardial. At baseline, older age was associated with higher volumes of inguinal, perinephric and pericardial WAT depots (Fig. 1H, K, and L). Young males had the highest volume of gonadal WAT at baseline (Fig. 1I).

In summary, older age was associated with lower rates of body weight and fat mass loss. Conversely, older age was associated with higher total fat mass at baseline and at the end of the study. Older age was also associated with higher volumes of inguinal, perinephric and pericardial WAT.

### Energy intake patterns and their relationship with sex and age

**Fig. 2:**
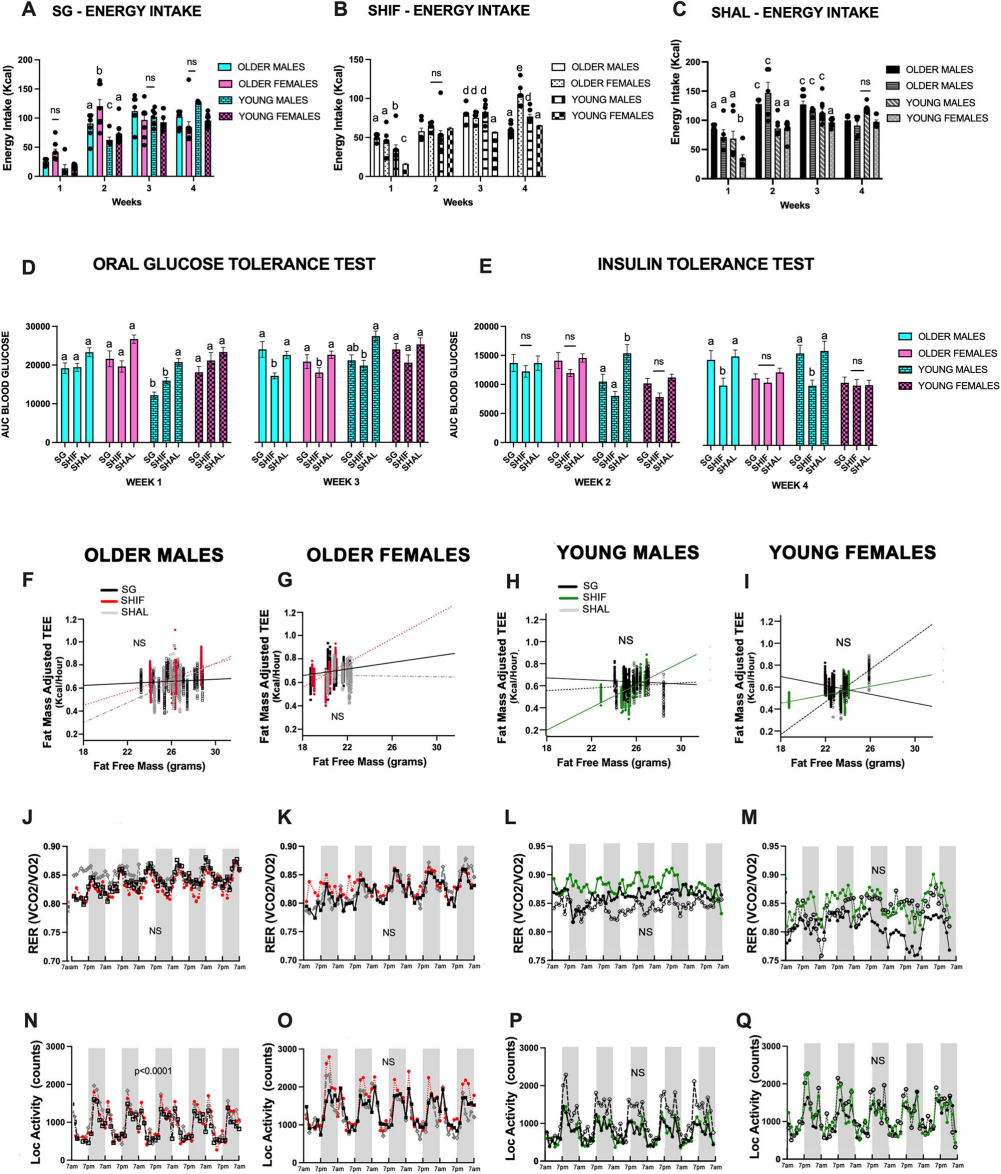
Energy intake, glucose homeostasis, energy expenditure and locomotor activity for SG, SHIF and SHAL mice after surgery. **A–C** Energy intake (EI) after SG, SHIF and SHAL, Two-Way ANOVA with multiple comparisons using Tukey’s tests. **A** For SG, there were differences in EI on weeks 1, 3 and 4. On week 2, older females had the highest EI (a versus b, *p* < 0.01; a versus c, *p* < 0.05). **B** For SHIF, on weeks 1 and 3, young females required the most food restriction to maintain the same body weight as young SG females (a versus b, *p* < 0.05; a versus c, *p* < 0.0001; a versus d, *p* < 0.01). On week 4, older females consumed the most calories while maintaining a similar body weight as SG females (a versus e, *p* < 0.0001). **C** For SHAL, young females had the lowest EI on weeks 1 and 3 (a versus b, *p* < 0.0001 and a versus c, *p* < 0.0001); on week 2, young males and females consumed less calories than their older counterparts. There was no difference among the groups on week 4. **D**, **E** Glucose homeostasis after SG, SHIF and SHAL. Two-Way ANOVA with Tukey’s tests for multiple comparisons. D. SG and SHIF young males had the best glucose tolerance on week 1 (a versus b, *p* < 0.01). On week 3, SHIF mice, except for young females, had the best performance on the OGTT. **E** ITT on week 2: Young SG and SHIF males performed better than young SHAL on week 2 (a versus b, *p* < 0.01). On week 4, older male and young male SHIF had improved insulin sensitivity compared to SG and SHAL, within each respective group (a versus b, *p* < 0.05). **2F–I** Total Energy Expenditure (TEE) adjusted for Fat-Free Mass (FFM) and Fat Mass (FM). No difference within any of the groups. **2J–M** Respiratory Exchange Ratio (RER). No difference within any of the groups**. 2N–Q** Locomotor Activity. Older SG males had the lowest activity level (*p* < 0.0001). Statistics:*N* = 6–14 per group; Two-Way ANOVA with multiple comparisons for energy intake and glucose homeostasis; Mann-Whitney for comparisons between two groups; Generalized Linear Regression with adjustment for FFM and FM for TEE and for RER; Two-Way ANOVA with mixed effects for locomotor activity. NS nonsignificant.

Unlike the clearly demarcated differences in SG weight loss outcomes associated with age, the impact of age on energy intake was not as prominent (Fig. 2A). In the first, third and fourth post-operative weeks, there were no differences in energy intake in the SG group. Older (middle-aged) male and female SG mice had a higher energy intake than younger ones on week 2.

What was notable in the SHIF group is that young females required the most strict caloric restriction to maintain a body weight similar to young females in the SG group (Fig. 2B). However, mice in the SHIF groups were on a calorie restriction of at least 20%, with adjustments made to keep SHIF mice’s weight matched to the SG groups. Lastly, age partially emerged as a factor influencing food intake in the SHAL cohort, as Older (middle-aged) mice generally consumed more calories than younger mice in weeks 1 and 2 (Fig. 2C).

### The influence of sex and age on glucose regulation after weight loss

Both glucose tolerance and insulin sensitivity in the first two post-operative weeks mirrored the pattern observed in energy intake: older (middle-aged) SG mice consumed the most energy and also had poor blood glucose outcomes on the OGTT and ITT (Fig. 2D, E). After those initial weeks, young males were the only SG group that continued to have improved blood glucose levels on the OGTT, similarly to SHIF mice (Fig. 2D). Older (middle-aged) SG mice and young female SG mice did not differ from SHAL mice in their OGTT performance during week 3 (Fig. 2D). Additionally, by the end of the study, SHIF mice continued to have improved insulin sensitivity compared to SG and SHAL, except for the young female group, where there were no discernible differences among SG, SHIF and SHAL mice (Fig. 2E).

### The influence of sex and age on energetics during weight loss

We also investigated if sex and age had an impact on EE, RER and locomotor activity (Fig. 2F–Q) and whether these differences could contribute to weight loss and glycemic outcomes. EE and RER were not significantly different for SG relative to SHIF and SHAL, in both the older (middle-aged) and young cohorts, irrespective of sex. The only group that showed a significant difference for locomotor activity was older (middle-aged) SG males, which had lower activity level compared to SHIF and SHAL mice in the same cohort (Fig. 2N–Q).

### WAT depot volume loss according to sex and age after SG and SHIF

**Fig. 3:**
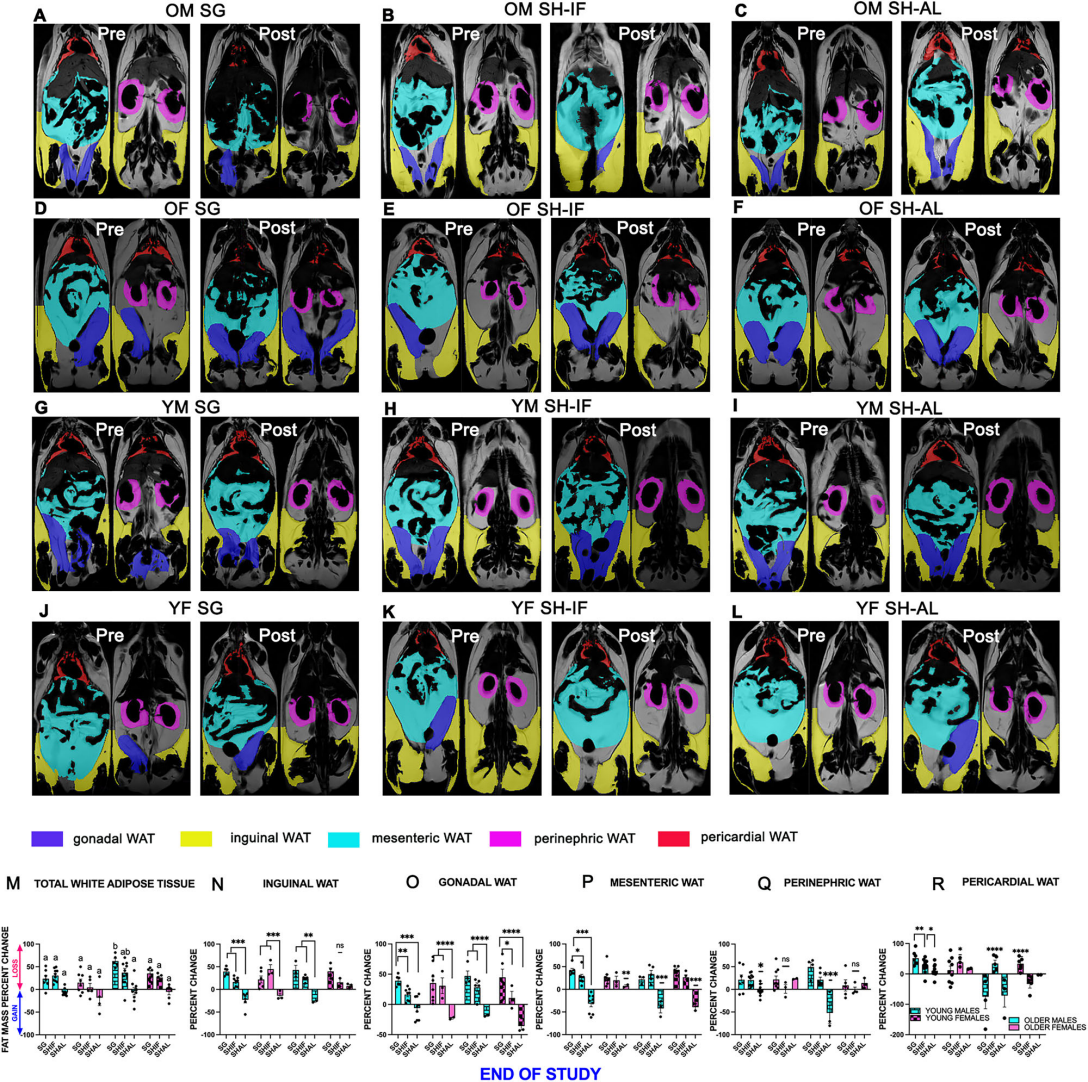
WAT depot volumes measured by MRI and percent change before and after SG, SHIF and SHAL surgery. **A–L** WAT depot volumes measured by MRI before and 4 weeks after SG, SHIF or SHAL in older males, older females, young males and young females. **3** **M** Total WAT percent change after SG, SHIF or SHAL in older males, older females, young males and young females. Two-Way ANOVA with Tukey’s multiple comparisons test (a versus b, *p* < 0.05). **3N–R** Percent change in iWAT, gWAT, mWAT, rWAT and cWAT after SG, SHIF or SHAL in older males, older females, young males and young females. *N* = 3–5 per group; Two-Way ANOVA with multiple comparisons; *p < 0.05, **p < 0.01, ***p < 0.001, ****p < 0.0001; ns nonsignificant.

We examined whether sex and age were associated with total WAT loss (Fig. 3M). We found that younger male SG mice experienced greater total WAT loss than the other groups(*p* < 0.05). Next, we used MRI scanning to measure the volume of specific WAT depots at baseline and then 4 weeks post-surgery (Fig. 3A–L). There was no difference among the 4 groups – older (middle-aged) males, older (middle-aged) females, young males and young females - in the pattern of WAT loss in response to SG or SHIF (Fig. 3N–R). SG generally produced the highest volume loss for iWAT, gWAT, mWAT and rWAT at about 40% loss (no overall statistical difference among SG groups). The only exceptions were older (middle-aged) SG females, who lost less mWAT, rWAT and cWAT and young SG males, who lost significantly less mWAT and cWAT compared to the other groups (Fig. 3N, P and R). Young males in the SG and SHIF groups lost significantly more iWAT than the SHAL group (Fig. 3N). There was no difference in the loss of iWAT among the 3 groups in the young female cohort, which was associated with high variability in their individual responses to SG and SHIF (Fig. 3N). Lastly, young female SHIF mice lost less gWAT, rWAT and cWAT (Fig. 3O, Q and R).

SHIF mice, in general, had the second highest loss of iWAT, gWAT and mWAT after SG, without differences between older (middle-aged) and younger mice (Fig. 3N, O and P). SHAL mice invariably gained WAT, except for young females, which lost iWAT, and older (middle-aged) females, which lost mWAT (Fig. 3O and Q). In summary, SG produced the most WAT loss, especially in young males, with the exception of mWAT.

### WAT transcriptome profiling according to sex and age

We examined the transcriptome of iWAT, gWAT and mWAT of older (middle-aged) and young mice that were randomized to the SG, SHIF and SHAL groups. We were unable to obtain enough tissue to isolate RNA from rWAT and cWAT that would pass the stringent quality control at our genomic center.

**Fig. 4:**
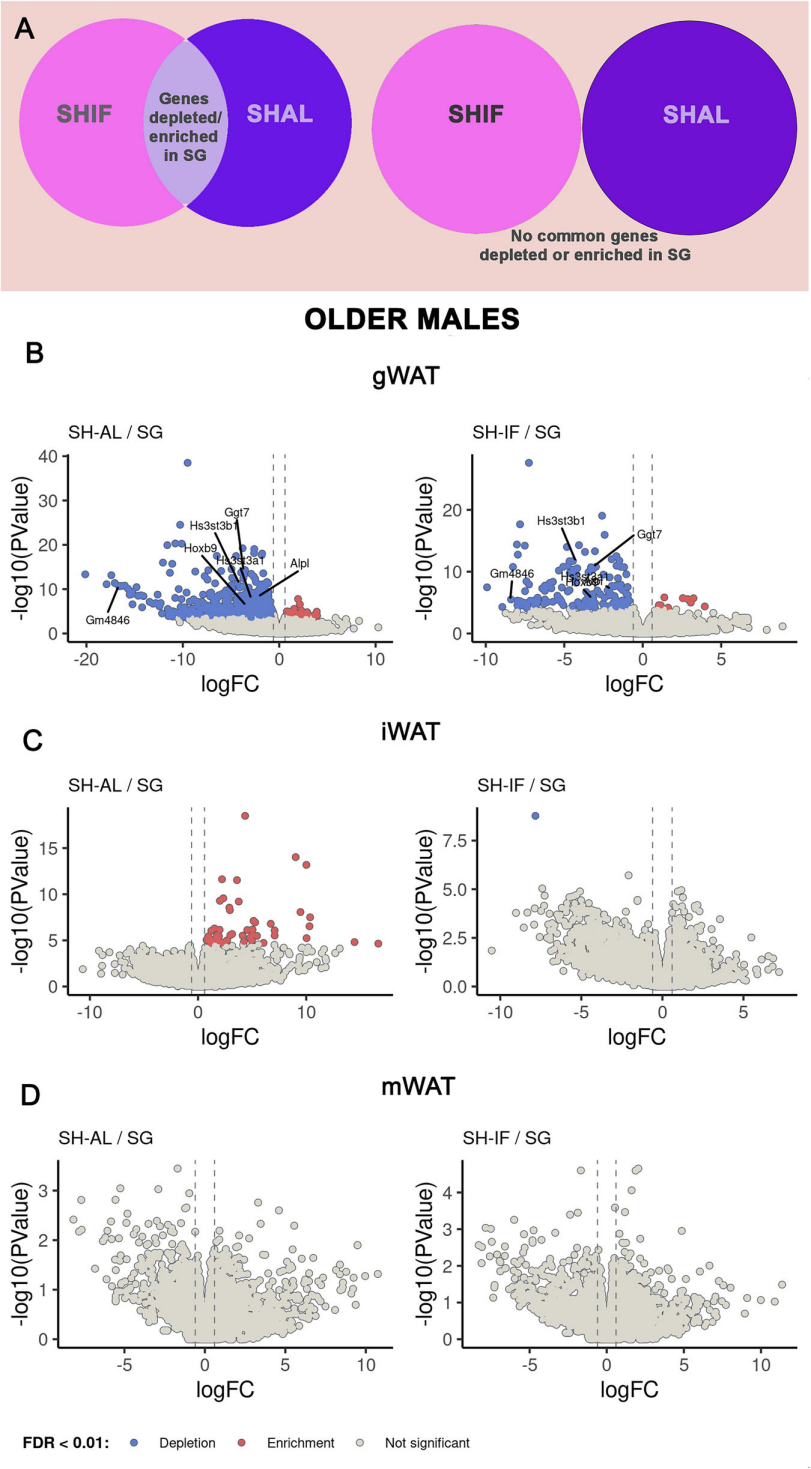
RNASeq data for different white adipose tissue types after surgery in older males. **A** Schematic representing the strategy used for analyzing the RNA sequencing data. We considered statistically significant genes with an FDR < 0.01, which were depleted or enriched in the SG group in relationship to both SHIF and SHAL groups simultaneously. **B–D** Volcano plots of RNAseq data in gWAT, iWAT and mWAT for older males.

We hypothesized that significant transcriptomic changes involving relevant genes for adipose tissue function would be associated with better metabolic outcomes from SG. As SG produces the most pronounced weight loss, our analysis was based on comparing genes with FDR < 0.01 and fold change ≥2 in mRNA expression in SG compared to SHIF and SHAL (Fig. 4A). All genes reported in this study are genes with confirmed expression in WAT, although the function of some of them is unknown.

In older (middle-aged) males, a few genes were significantly depleted in the gWAT of SG mice compared to SHIF and SHAL mice: Hs3st3b1, Hs3st3a1, Ggt7, Gm4846, Hoxb9, Alpl (Fig. 4B). In particular, Hs3st3b1 and Hs3st3a1 are involved in glycosaminoglycan biosynthesis and heparan sulfate-glucosamine 3-sulfotransferase 1 activity in WAT^25^. Leanness is associated with lower expression of heparan sulfates in the extracellular matrix of WAT^26^. Ggt7 is involved in glutathione biosynthesis, Gm4846 in lipid metabolism, Hoxb9 in cell cycle regulation and Alpl in lipid metabolism and secretion of adipokines^27–30^. Other than gWAT, there were no significantly depleted or enriched genes in the iWAT and mWAT of older (middle-aged) males (Fig. 4C and D).

**Fig. 5:**
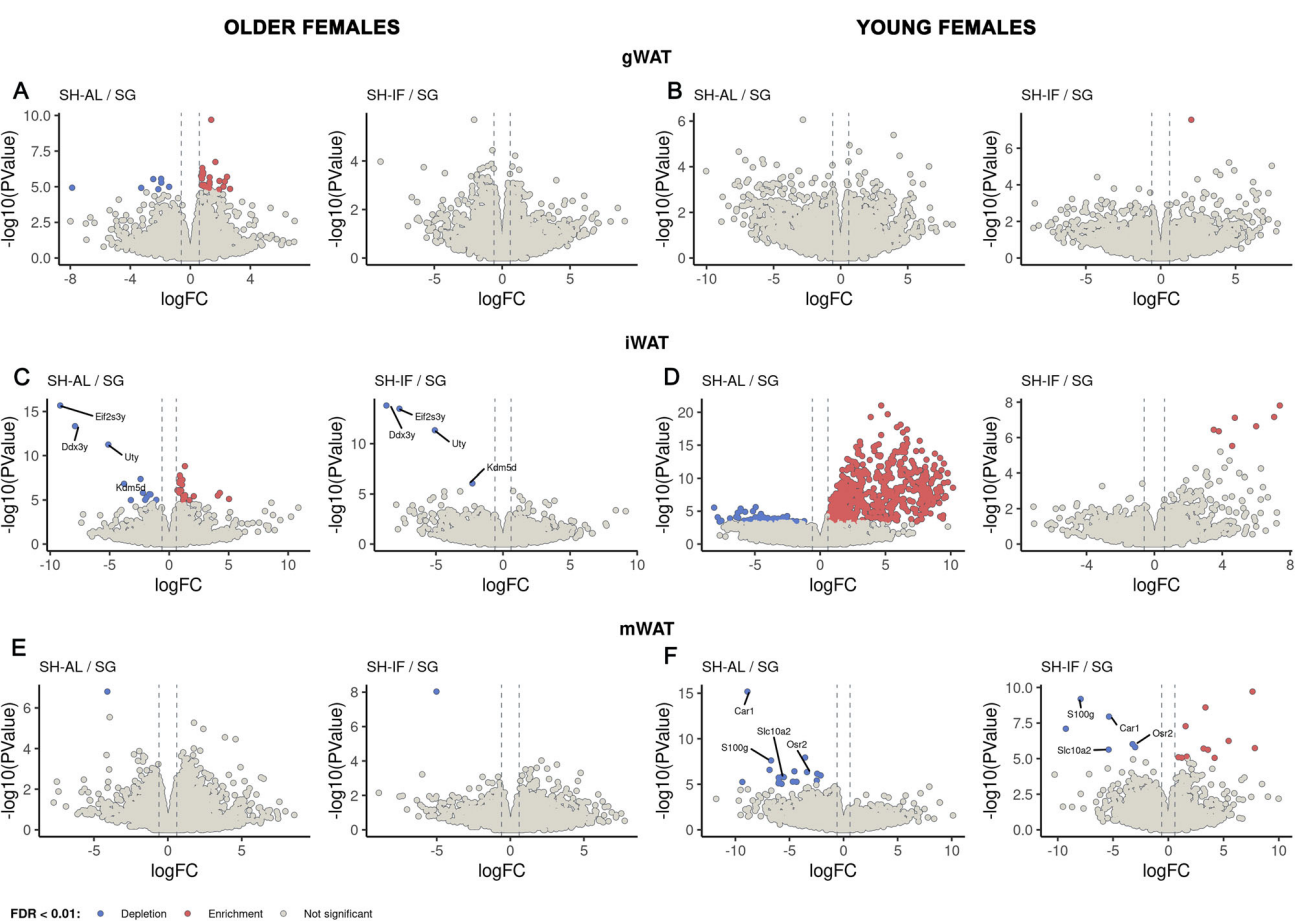
RNASeq data for different white adipose tissue types after surgery in older and young females. **A**, **C**, **E** Volcano plots of RNAseq data in gWAT, iWAT and mWAT for older females. **B**, **D**, **F** Volcano plots of the RNAseq data in gWAT, iWAT and mWAT for young females.

No genes met the above criteria in the SG/SHAL and SG/SHIF comparison for gWAT and mWAT in older (middle-aged) females or for gWAT and iWAT in young females (Fig. 5A, E, B, and D). The transcriptome analysis of iWAT in older (middle-aged) females revealed the following genes that were significantly depleted in SG compared to SHAL/SHIF: Eif2s3x, Ddx3x, Utx, and Kdm5 (please see supplemental data for lists of all genes with FDR, p value, and fold change) (Fig. 5C). These genes are X-inactivation escape genes which are key for determining characteristics that define sex dimorphism^31^. Genes that were significantly depleted in the mWAT of young females were S100g, Slc10a2, Car1 and Osr2. S100g is part of the S100 protein family linked to macrophage-induced inflammation (Fig. 5F)^32^. Slc10a2 is a bile acid transporter that is expressed in mammalian WAT^33^. Car1 encodes a cytoplasmic carbonic anhydrase ubiquitously expressed^34^, while Osr2 is expressed in adipocyte progenitors^35^.

**Fig. 6:**
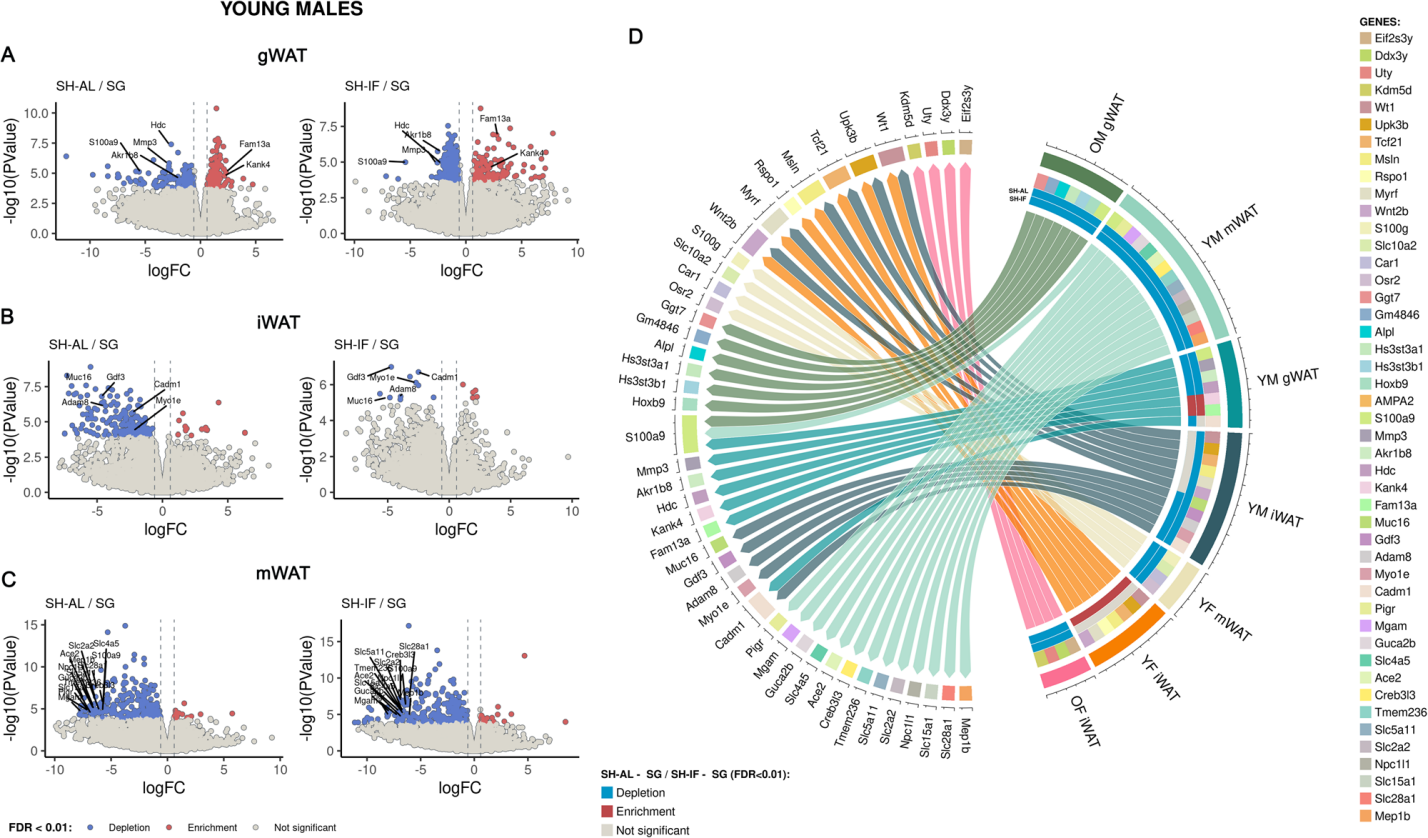
RNASeq data for different white adipose tissue types after surgery in young males. **A**–**C** Volcano plots of the RNAseq data in gWAT, iWAT and mWAT for young males. **D** Chord diagram showing relationships between SG gWAT, iWAT and mWAT in older males, older females, young males and young females and differential gene expression.

Young males uniquely expressed genes that were either significantly enriched or depleted in SG compared to SHIF/SHAL, in all 3 WAT depots. For example, in gWAT, genes S100a9, Mmp3, Hdc and Akr1b8 were significantly depleted in SG (Fig. 6A). S100a9 and Mmp3 are part of the IL-17 signaling pathway and innate immune response, while Hdc is involved in histamine signaling^36–39^. Ark1b8 is believed to be an ortholog of human Akr1B10, an aldo reductase involved in the regulation of fatty acid synthesis^40^. On the other hand, genes Fam13a and Kank4 were enriched in the gWAT of young male SG mice (Fig. 6A). Fam13a is involved in regulation of adipocyte differentiation and Kank4 is expressed in endothelial muscle^41,42^.

The iWAT of SG mice in the young male cohort exhibited a significant depletion of the following genes: Gdf3, Adam8, Myo1e, Muc16 and Cadm1 (Fig. 6B). Adam8, Myo1e and Cadm1 are involved in leukocyte migration during inflammation, positive regulation of cytokine production and vasculogenesis^43–46^. Muc16 (or CA125) is the largest transmembrane mucin and is highly expressed in WAT^34,47^.

Genes that were depleted in the mWAT of young SG males were Slc28a1, Slc5a11, Slc15a1, Slc2a2, Slc4a5, S100a9, ACE2, Npc1l1, Mgam, Creb3l3, Guca2b, Pigr, Mep1b and Tmem236 (Fig. 6C). These genes are expressed in WAT and have diverse roles in cholesterol metabolism, inflammatory response and leukocyte migration (IL-17 signaling pathway), virus receptor activity, among others (please see Discussion section).

In conclusion, only young males displayed significant transcriptomic changes in iWAT, gWAT and mWAT after SG. Young females did not show significant transcriptome changes except for mWAT, while older (middle-aged) females and males displayed changes in gWAT and iWAT, respectively.

We were able to observe convergence of a pattern of expression among similar tissues or within a specific experimental group. For example, SG led to the depletion of S100a9 in the gWAT of both older (middle-aged) and young males (Fig. 6D). SG also caused the depletion of S100a9 in the mWAT of young males. Cadm1 was depleted in the iWAT and gWAT of young males. The group of genes that included Wt1, Upk3b, Tcf21, Msln, Rspo1, Myrf and Wt2b were enriched in SG relative to SHIF in the young female iWAT were nonsignificantly depleted in young male iWAT relative to SHIF. At least for this group of genes, iWAT in young females and males had an inverse pattern of expression.

## Discussion

SG and IF are weight loss methods frequently prescribed by health care professionals to individuals who are obese and are not candidates for GLP-1RA use. There are a number of significant sex- and age-specific differences in adipose tissue biology and distribution that make it important to know whether sex and age influence the outcomes of weight loss treatments. Some of the sex differences stem from Y versus X chromosome effects, the differential expression of lipoprotein lipase in adipose tissue, the differential expression of lipogenic versus lipolytic adrenergic receptors and the influence of sex hormones^48,49^. As far as differences produced by aging, they include adipocyte dysfunction through the release of pro-inflammatory cytokines, aberrant adipogenesis, increased fibrosis and immune cell infiltration^50^. While the results of our study cannot be directly translated to human clinical care, the findings are compelling enough to bring our attention to the influence of sex and age on weight loss therapeutic outcomes in human trials.

First, we showed that older age is unquestionably associated with lower weight loss after SG or IF, irrespective of sex. Although older (middle-aged) mice had higher total WAT mass and higher volumes of iWAT, gWAT, rWAT and cWAT at baseline, they nonetheless lost less WAT and by the study’s end, still had more fat mass than younger mice. Secondly, we found that being female was associated with a lower total WAT loss and a lack of uniformity in the loss of specific WAT depots after sleeve gastrectomy (SG) or sham surgery with intermittent fasting (SHIF), with individual responses showing high variability. Additionally, we demonstrated that young males that underwent SG or SHIF had the best performance on the OGTT and ITT. As a result, the study suggests that younger age and male sex are advantageous biological traits for weight loss and glycemic outcomes after SG or SHIF. The findings in older (middle-aged) females were not a result of loss of estrous cycles, as they maintained them, as we previously demonstrated in females of similar age^51^.

We did not find any differences in energy intake, locomotor activity or energy expenditure that could explain our results. What we found is that the best metabolic outcomes, which were observed in younger males, were associated with a higher degree of transcriptome plasticity in iWAT, gWAT and mWAT, unlike what was observed in other groups. More importantly, the WAT transcriptomic changes observed in young males involved genes that are relevant for adipose tissue function.

The main transcriptomic changes in gWAT and mWAT of young males appeared to have a bias towards a reduction in inflammatory processes, as evidenced by the depletion of S1009a and Mmp3, which are part of the IL-17 signaling pathway^36–38^. While the mRNA expression pattern in the iWAT of young males also involved the depletion of genes involved in inflammatory responses, there were also other notable findings in this group, namely the depletion of Gdf3 and Cadm1. Growth differentiation factor 3 (Gdf3) is a protein in the TGFβ superfamily and found in increased levels in obesity, aging and inflammation^46^. It has been proposed that Gdf3 loss of function in mouse WAT leads to a reduction in body weight and improved insulin sensitivity^46^. In addition, there is evidence that downregulation of Cadm1 promotes insulin sensitivity and decreases inflammation in WAT^44^.

Based on pathway analysis utilizing DAVID^52,53^, we hypothesize that one consequence of the depletion of Slc2a2, Slc5a11, Mgam, Npc1l1, Slc28a1, Slc15a1 and Slc4a5 in mWAT, as seen in young males, would be a deceleration in lipid and carbohydrate transport and metabolism^54–60^. How this could contribute to improved weight loss and glucose homeostasis is unclear.

Another noteworthy finding was a reduction in the mRNA expression of ACE2 in the mWAT of young males, similarly to the finding that RYGB in human patients downregulates ACE2 expression in subcutaneous WAT (in that study, visceral WAT was not tested)^61^. Binding of the coronavirus to ACE2 is a critical step in SARS-CoV-2 infection^62^. When human patients with obesity lose weight, there is a decrease in the expression of ACE2 in subcutaneous WAT^63^. While ACE2 is highly expressed in human visceral and subcutaneous WAT, it is not known if a reduction in ACE2 expression in WAT decreases one’s risk of acquiring severe SARS-CoV-2 infection^64^. The other two genes depleted in the mWAT of young males, Creb3l3 and Guca2b suggest that adipose tissue development is not favored after SG^65,66^. Fam13a and Kank4 were the only genes significantly enriched in young male gWAT. Fam13a is highly expressed in WAT and has been linked to adipocyte differentiation and distribution^41^. In one study, the Kank4 locus in humans was positively associated with one’s ability to lose weight while undergoing a low-calorie diet^67^.

It was intriguing that in older (middle-aged) females, iWAT transcriptomic changes after SG involved the depletion of Eif2s3x, Ddx3x, Utx, and Kdm5, which are genes associated with the phenotypic expression of female-specific characteristics. This suggests that a more effective metabolic response to SG, characterized by greater weight loss, may depend on the inhibition of signaling pathways upon which the expression of female traits depend. Especially in the case of Kdm5, its mRNA expression has been found to be positively associated with body mass^68^.

Our study found no differences in the pattern of redistribution of WAT depots after SG or SHIF, regardless of age or sex. SG produced the greatest loss of WAT, except for mWAT and cWAT in younger males. Although accumulation of human visceral adiposity is considered a risk factor for cardiovascular and metabolic diseases^69,70^, the more limited mWAT loss observed in young males did not jeopardize their favorable metabolic response to SG. Our study suggests that mWAT function is more important than its overall size or burden, as transcriptomic changes induced by SG may have potentially led to decreased inflammation and a healthier mWAT. On the other hand, young males had the greatest loss of perinephric WAT, which is considered a type of visceral adipose tissue. This loss could have potentially compensated for the limited loss of mWAT in young males. Whether this is correlated in any way to the improved metabolic outcomes in young males needs to be explored by future studies.

One potential limitation of our study is the fact that females were reared in our laboratory from age 6 weeks, whereas males were purchased between ages 10-12 weeks. This reflects the fact that female DIO mice are not commercially available and had to be developed by ourselves. Whereas these differences in rearing for a few weeks contributed to the results is not known. One other potential limitation in the glucose regulation studies is that during the first week post-operatively, mice are on a liquid and solid diet, whereas they only receive solid food afterwards. The presence of control groups with different treatments but exactly the same type of diet is likely to have eliminated this potential source of bias.

Finally, one question that begs to be answered is whether unfavorable biological traits can be modified in the pursuit of improved metabolic health. Could the metabolic outcomes of SG or IF be improved in older (middle-aged) mice by attenuating senescent traits through exercise, a specific diet composition or pharmacological agents?

## Methods

### Animals, housing and experimental design

The studies were performed according to ARRIVE guidelines and in accordance with the Columbia University Institutional Animal Care and Use Committee, which granted approval for all the experiments in this study. Female C57bl/6 J mice were purchased from The Jackson Laboratories at 6 weeks of age and maintained thereafter on a 60% high fat diet (Research Diets, catalogue number D12492, New Jersey, NJ). C57bl/6 J obese female mice are not commercially available. Male C57bl/6 J raised on a 60% high fat diet from 6 weeks of age, were purchased from The Jackson Laboratories at 10 to 12 weeks of age and maintained on the same 60% high fat diet as the female mice throughout all studies. Mice were individually housed in a vivarium maintained at 22 °C on a 12:12 (7 am - 7 pm) light / (7 pm - 7 am) dark cycle. The mice for the young cohorts were randomized at 22 weeks of age, and the ones for the older (middle-aged) cohorts at 55–60 weeks of age to sleeve gastrectomy (SG), sham surgery with intermittent fasting (SHIF) weight matched to the SG group and sham surgery with ad libitum feeding (SHAL). Intermittent fasting (IF) involved feeding the SHIF group a variable ration, once daily in the early morning, on weekdays, with the amount depending on their weight loss or gain, so that they would be weight-matched to the SG group. From Fridays to Mondays, SHIF mice were allowed to eat without restriction (*ad libitum*). Baseline measurements were defined as having been taken 2 weeks prior to surgeries. Body weight (BW) and food intake were measured daily for the first week post-operatively and weekly thereafter. All cohorts were sacrificed 4 weeks post-operatively, after an overnight fast, under isoflurane anesthesia at surgical plane, for tissue harvest. Four weeks for mice are equivalent to 25 to 45 months in human time depending on the mice’s age^71^. Inguinal white adipose tissue (iWAT), gonadal white adipose tissue (gWAT), mesenteric white adipose tissue (mWAT) and the gastrocnemius muscle were sampled for mRNA analysis. Weight loss, body composition, food intake and glucose homeostasis studies were replicated for all cohorts.

### Sleeve gastrectomy

All surgical procedures were sterile, and all surgeries were performed by the same surgeon. Mice received meloxicam (Meloxicam, Boehringer Ingelheim, 5 mg/Kg), saline and enrofloxacin (Enrofloxacin, Bayer, 5 mg/Kg) subcutaneously at the time of the surgery. The SG surgery described here is a modification of a previously published SG surgical technique [25]. Animals were not fasted overnight. Surgeries were performed under a dissecting microscope (Leica M-125). A midline laparotomy was performed and the stomach isolated from surrounding connective tissue. Then the gastric arteries were ligated using 8-0 suture (DemeTECH DemeCRYL 6.5 mm micropoint curved, eSutures, G1880065G7P). Thereafter, an incision was performed at the bottom of the fundus to empty the stomach and irrigate it with warm saline. Next, an imaginary line was traced from 2 mm below the esophagogastric junction to the point where the pancreas is attached to the stomach. All gastric tissue below that line was dissected with microscissors, then the edges of the stomach at the line of resection were sutured together using 7-0 suture (DemeTECH DemeCRYL 6.5 mm micropoint curved, eSutures, G1870065G7P). Gastric leaks were assessed, then the abdominal wall was closed in layers using 5-0 suture (Vicryl Violet, eSutures, J303). Mice were maintained on a liquid diet with Ensure High Protein Vanilla (Abbott Laboratories) and high fat diet (Research Diets, D#12492) for 4 days prior to the surgeries. The day before the surgery, high fat diet was removed and the mice were kept on Ensure until the 7th post-operative day overlapping with the reintroduction of high fat diet on the 5th post-operative day. The high fat diet was then continued until the end of the study. After the first week post-surgery, SG and SHAL groups were kept on ad libitum high fat diet. The SHIF group was kept on approximately 1.0–2.5 grams of high fat diet daily, provided at different times of the day to limit entrainment. On weekends, SHIF mice were fed ad libitum.

### Glucose tolerance test and insulin tolerance test

Mice were fasted for 6 h prior to glucose and insulin tolerance tests. SHIF mice were fed from 0.8–1.0 g of high fat diet one hour prior to the start of the 6 h fast, in preparation for the glucose and insulin tolerance tests. This was done to avoid a more prolonged period of fasting in SHIF mice, compared to SG and SHAL mice. Oral glucose tolerance tests (OGTT) were performed at 1 and 3 weeks post-operatively; insulin tolerance tests (ITT) were performed 2 and 4 weeks post-operatively. OGTT were performed by oral gavage using a plastic feeding tube (1FTP-20-38, Instech Laboratories, Inc.), with 2 grams of dextrose per kg of body weight. Insulin tolerance tests ITT were performed using regular insulin at a dose of 0.75 units per Kg of body weight, injected intraperitoneally (Humulin R, Lilly).

### Body composition

Body composition was measured at baseline, and at four weeks post-operatively using Echo-MRI^TM^-100H (EchoMRI LLC, Houston, TX).

### Energy expenditure and locomotor activity

Energy expenditure by indirect calorimetry was performed by singly housing mice in the Comprehensive Lab Animal Monitoring System (CLAMS, Columbus Instruments, Columbus, OH) for one week, at baseline (22 weeks for young mice; 55–60 weeks for older mice) and then 4 weeks post-operatively. The SH-IF group was fed ad libitum during the calorimetry experiment. CLAMS measurements included Volume O2, O2 in and O2 out, delta O2, Accumulated O2, Volume CO2, CO2 in and CO2 out, delta CO2, Accumulated CO2, RER, Heat, Flow, Feed Weight, Feed Accumulation, and Total Locomotor Activity, which was calculated as the sum of activity along the X, Y and Z axes of the chambers. For the 4-week time point total energy expenditure measurement, we used a fully validated method for estimating energy expenditure that accurately replicates findings from indirect calorimetry^72,73^. Analysis utilized Generalized Linear Regression by SPSS with adjustment for FFM and FM. The first two days of data were excluded as the mice were acclimating to the calorimetry chambers. Analysis included 5 entire days of data, with selection of the maximum value within each hour, for each mouse. Graphs for Fig. 2F–I were generated by SPSS.

### RNA extraction

Inguinal, gonadal, and mesenteric fat pads were dissected and processed for RNA extraction using RNeasy® Mini Kit (Qiagen, catalogue #74106). There were 4 biological replicates for each experimental group (SG, SHIF, SHAL in young male and young female cohorts, and older (middle-aged) male and older (middle-aged) female cohorts) for each fat pad (gWAT, iWAT and mWAT) for a total of 192 separate samples. RNA Integrity Number was greater than 8 for all samples. Samples from rWAT and cWAT were not processed due to low RNA content. RNA sequencing was performed by the JP Sulzberger Columbia Genome Center using STRPOLYA TruSeq Stranded mRNA Library Prep Kit (Illumina) paired end with 20 million reads. Files were generated as raw fastq files, and kallisto was used for quantifying abundances of transcripts^74^.

DAVID was used for pathway analysis.

### RNAseq analysis

The RNA sequencing analysis was performed using packages from the Bioconductor network of R software (version 4.4.0), specifically employing the edgeR package^75^. The visualization of the results was conducted using the ggplot2 and ggpubr packages. In the process of analyzing differential genes, an initial data filtering process was undertaken to remove anomalous values, such as genes with zero counts or genes of reduced length. These genes were disregarded due to their low expression, which prevents them from achieving statistical significance. Subsequently, data normalization was carried out, transforming sample information to a common scale, and minimizing data noise to render them comparable. Finally, the analysis of differential expression was conducted using the exact Test function, based on the negative binomial model, which enables pairwise analysis of differential gene expression. The analyzed genes were considered differentially expressed if the false discovery rate (FDR) was less than 0.01 and a fold change >2 or <-2, with these results presented in volcano plots.

### MRI methods

The MRI experiments were performed on a Bruker BioSpec 9.4 Tesla preclinical scanner operating on Paravision (PV.7.0.0) software platform (Bruker Corp., Billerica, MA). The mice were anesthetized with 1–2% isofluorane mixed with medical air via a nose cone. The concentration of the isoflurane was adjusted during the procedure to maintain the respiration rate in the range of 40–70 breaths/min using a respiration pillow attached to a monitoring system (SA Instruments, Stony Brook, NY). Animals were placed prone on the animal bed inside a circular polarized birdcage coil with an inner diameter of 72 mm. The images were acquired using Rapid Acquisition with Relaxation Enhancement (RPRE) sequence with fat-water separation technique with the following parameters: TR = 1448 ms, TE = 26 ms, Resolution=265 × 265 μm^2^. Coronal images with slice thickness of 1.2 mm were obtained with 24 slices to cover the animal’s entire body. An angle of 140° between fat and water magnetization was chosen for out of phase image. Two separate images of fat and water were generated, respectively. Additionally, a re-combined fat-water image was reconstructed which showed the mouse’s anatomy clearly.

### MRI analysis

The adipose tissue depots from the high-quality MRIs were subsequently analyzed using the Analyze Direct 14.0 software (Analyze Direct, KS, USA). Images were sorted by cohorts and further into pre- and post-interventions. No preferences were given to sorting groups based on a specific intervention during the splicing to reduce biases. Lab members, who were blinded for the interventions, manually captured adipose tissue depots surrounding the pericardial, perinephric, perigonadal, mesenteric WAT, and subcutaneous tissues using the spline trace and auto-trace features within the software. Determinations of the adipose boundaries were adapted from illustrations by Margaret J. Cook in ‘The Anatomy of the Laboratory Mouse’ and the work of Bagchi & MacDougald^76,77^. Post splicing, the groups were sorted based on specific experimental interventions. The number of pixels for each tissue depot was multiplied by voxel width, voxel height, and voxel depth for each slice and computed together by the Analyze 14.0 software to obtain each depot’s volume. All the volumes were later transferred into an Excel file (Microsoft Excel, Redmond, WA, USA). For each mouse, per fat depot, the difference between the pre-intervention and post-intervention volumes was calculated. Fat depots for mice from the same intervention group were averaged and compared against the mice from other intervention groups.

### Statistics

We used GraphPad Prism 10 version software. Two-way ANOVA with Multiple Comparisons with Tukey tests were used for analysis of multiple groups involving repeated measures. One-way ANOVA with Tukey tests were used for comparisons of single measures among more than two groups. Data are expressed as means ± SEM. Area Under the Curve (AUC) was generated for OGTT and ITT and then compared using One-way ANOVA with Multiple Comparisons and Tukey tests. Column factor refers to surgical group and row factor to time. For all analyses, power was set at 80%, with a *p* value of <0.05. Linear Regression was used to analyze calorimetry data.

## Supplementary information

Supplementary Material

## Data Availability

All data is contained within the manuscript and supplementary files, and the full raw dataset is available in Figshare (10.6084/m9.figshare.28689224). Link for Figshare: https://figshare.com/s/277368739fa06a01b8d3.
