# Supplementary material for: The metabolic advantage of being young and male in obesity treatment outcomes in mice
Source: NPJ Metab Health Dis. 2025 Aug 1;3:32. doi: 10.1038/s44324-025-00065-2 (PMC12316924; doi:10.1038/s44324-025-00065-2)
Supplement: Supplementary file 1 — Supplementary Material [file 44324_2025_65_MOESM1_ESM.pdf]

S U P P L E M E N T A R Y   M A T E R I A L:

The metabolic advantage of being young and male for obesity treatment outcomes in mice

Amanda Dirnberger, Elen Yanina Aguirre-Rodriguez, Elias Carlos Aguirre-Rodriguez, John O. Degraft Hanson, Yanping Sun, Dave Delima, Benjamin F. Bykov, Aneirson F. Da Silva, Marko Kraljevic, Fernando AS Marins, Ana Emiliano

```
library(readr)
library(DESeq2)
library(edgeR)

df <- read.delim("est_counts_genes_kallisto.txt")
Group <- read.csv('dfGroup.csv', header=TRUE)
DGEList <- DGEList(counts=df,group=factor(Group[,7]))
Filt <- filterByExpr(DGEList)
#Gene filtering.
DGEList <- DGEList[Filt, , keep.lib.sizes=FALSE]
#Normalizing the data.
DGEList <- normLibSizes(DGEList)
#First, the common dispersion (total variability) is estimated, followed by the
#estimation of tagwise dispersion (gene-to-gene dispersion).
condition_ <- DGEList$samples$group
DGEList_d <- estimateCommonDisp(DGEList,design = model.matrix(~condition_), verbose=T)
```

```
## Disp = 0.15466 , BCV = 0.3933
```

```
DGEList_d <- estimateTagwiseDisp(DGEList_d,design = model.matrix(~condition_))
```

```
mFun <- function(df,pair,lista,title){
  result <- exactTest(df, pair=pair)

  write.csv(topTags(result, n=500), paste(paste(pair,collapse=" "),".csv",
                                          sep=""), row.names=TRUE)
  dec.result <- decideTests(result, adjust.method="BH", p.value=0.01)
}
```

```
#read.csv(paste(paste(pair,collapse=" "),".csv",sep=""))
list_par_SG_SHAL <- list(c("SG.iWAT.Old_females", "SH-AL.iWAT.Old_females"),
                         c("SG.gWAT.Old_females", "SH-AL.gWAT.Old_females"),
                         c("SG.mWAT.Old_females", "SH-AL.mWAT.Old_females"),
                         c("SG.iWAT.Young_females", "SH-AL.iWAT.Young_females"),
                         c("SG.gWAT.Young_females", "SH-AL.gWAT.Young_females"),
                         c("SG.mWAT.Young_females", "SH-AL.mWAT.Young_females"),
                         c("SG.iWAT.Young_males", "SH-AL.iWAT.Young_males"),
                         c("SG.gWAT.Young_males", "SH-AL.gWAT.Young_males"),
```

```
                          c("SG.mWAT.Young_males", "SH-AL.mWAT.Young_males"),
                          c("SG.iWAT.Old_males", "SH-AL.iWAT.Old_males"),
                          c("SG.gWAT.Old_males", "SH-AL.gWAT.Old_males"),
                          c("SG.mWAT.Old_males", "SH-AL.mWAT.Old_males"))

list_par_SG_SHIF <- list(c("SG.iWAT.Old_females", "SH-IF.iWAT.Old_females"),
                          c("SG.gWAT.Old_females", "SH-IF.gWAT.Old_females"),
                          c("SG.mWAT.Old_females", "SH-IF.mWAT.Old_females"),
                          c("SG.iWAT.Young_females", "SH-IF.iWAT.Young_females"),
                          c("SG.gWAT.Young_females", "SH-IF.gWAT.Young_females"),
                          c("SG.mWAT.Young_females", "SH-IF.mWAT.Young_females"),
                          c("SG.iWAT.Young_males", "SH-IF.iWAT.Young_males"),
                          c("SG.gWAT.Young_males", "SH-IF.gWAT.Young_males"),
                          c("SG.mWAT.Young_males", "SH-IF.mWAT.Young_males"),
                          c("SG.iWAT.Old_males", "SH-IF.iWAT.Old_males"),
                          c("SG.gWAT.Old_males", "SH-IF.gWAT.Old_males"),
                          c("SG.mWAT.Old_males", "SH-IF.mWAT.Old_males"))

for (i in 1:12) {
  mFun(DGEList_d,list_par_SG_SHAL[[i]],lista = 0, title = "SH-AL / SG")
  mFun(DGEList_d,list_par_SG_SHIF[[i]],lista = 0, title = "SH-IF / SG")
}
```

```
list_name <- list("OF iWAT", "OF gWAT", "OF mWAT",
                  "YF iWAT", "YF gWAT", "YF mWAT",
                  "YM iWAT", "YM gWAT", "YM mWAT",
                  "OM iWAT", "OM gWAT", "OM mWAT")

Genes <- c("Eif2s3y", "Ddx3y", "Uty", "Kdm5d", "Wt1", "Upk3b", "Tcf21",
           "Msln", "Rspo1", "Myrf", "Wnt2b", "S100g", "Slc10a2", "Car1",
           "Osr2", "Ggt7", "Gm4846", "Alpl", "Hs3st3a1", "Hs3st3b1", "Hoxb9",
           "AMPA2", "S100a9", "Mmp3", "Akr1b8", "Hdc","Kank4", "Fam13a",
           "Muc16", "Gdf3", "Adam8", "Myo1e", "Cadm1", "Pigr", "Mgam",
           "Guca2b", "Slc4a5", "Ace2", "Creb3l3", "Tmem236", "Slc5a11",
           "Slc2a2", "Npc1l1", "Slc15a1", "Slc28a1", "Mep1b")

NewList <- function(list_par, list_name, Genes){
  Dat_f <- data.frame(Genes = Genes)
  for (i in 1:12) {
    nm = "/home/elen/Documentos/Elen/1. UNESP/DOUTORADO/Projeto_Columbia/Chord Diagram/"
    dt <- read.csv(paste(nm,paste(list_par[[i]],collapse=" "),".csv",
                          sep=""))
    filter_dt <- dt[(dt$PValue < 0.01) & (dt$FDR < 0.01) &
                  (dt$logFC < -0.6 | dt$logFC > 0.6), ][, c("X","logFC")]

    if (nrow(filter_dt) == 0) {
       Dat_f[paste(list_name [[i]],collapse=" ")] <- 0
    } else {
      val_match <- numeric()
      for (j in 1:nrow( Dat_f)) {
        gn <-  Dat_f$Genes[j]
        new_df <- filter_dt[filter_dt$X == gn,]
        if (nrow(new_df) == 0) {
          val_match[j] <- 0
```

```r
      } else {
        val_match[j] <- new_df$logFC
      }
    }
    Dat_f[paste(list_name [[i]],collapse=" ")] <- val_match
  }
}
print(Dat_f)
}
```

```r
df_shal2 <- NewList(list_par = list_par_SG_SHAL, list_name = list_name,
                    Genes = Genes)
```

```
##       Genes   OF iWAT OF gWAT OF mWAT YF iWAT YF gWAT   YF mWAT   YM iWAT
## 1   Eif2s3y -9.155453       0       0       0       0  0.000000  0.000000
## 2     Ddx3y -7.908727       0       0       0       0  0.000000  0.000000
## 3       Uty -5.112635       0       0       0       0  0.000000  0.000000
## 4     Kdm5d -2.202709       0       0       0       0  0.000000  0.000000
## 5       Wt1  0.000000       0       0       0       0  0.000000 -5.462863
## 6     Upk3b  0.000000       0       0       0       0  0.000000 -5.786876
## 7     Tcf21  0.000000       0       0       0       0  0.000000 -4.485772
## 8      Msln  0.000000       0       0       0       0  0.000000 -6.946213
## 9     Rspo1  0.000000       0       0       0       0  0.000000  0.000000
## 10     Myrf  0.000000       0       0       0       0  0.000000 -3.560386
## 11    Wnt2b  0.000000       0       0       0       0  0.000000 -5.463639
## 12    S100g  0.000000       0       0       0       0 -6.700001  0.000000
## 13  Slc10a2  0.000000       0       0       0       0 -5.522383  0.000000
## 14     Car1  0.000000       0       0       0       0 -8.886033  0.000000
## 15     Osr2  0.000000       0       0       0       0 -3.378779  0.000000
## 16     Ggt7  0.000000       0       0       0       0  0.000000  0.000000
## 17   Gm4846  0.000000       0       0       0       0  0.000000  0.000000
## 18    Alpl   0.000000       0       0       0       0  0.000000  0.000000
## 19 Hs3st3a1  0.000000       0       0       0       0  0.000000  0.000000
## 20 Hs3st3b1  0.000000       0       0       0       0  0.000000  0.000000
## 21    Hoxb9  0.000000       0       0       0       0  0.000000  0.000000
## 22    AMPA2  0.000000       0       0       0       0  0.000000  0.000000
## 23   S100a9  0.000000       0       0       0       0  0.000000  0.000000
## 24     Mmp3  0.000000       0       0       0       0  0.000000  0.000000
## 25   Akr1b8  0.000000       0       0       0       0  0.000000  0.000000
## 26      Hdc  0.000000       0       0       0       0  0.000000  0.000000
## 27    Kank4  0.000000       0       0       0       0  0.000000  0.000000
## 28   Fam13a  0.000000       0       0       0       0  0.000000  0.000000
## 29    Muc16  0.000000       0       0       0       0  0.000000 -7.303877
## 30     Gdf3  0.000000       0       0       0       0  0.000000 -4.639438
## 31    Adam8  0.000000       0       0       0       0  0.000000 -4.344813
## 32    Myo1e  0.000000       0       0       0       0  0.000000 -2.193140
## 33    Cadm1  0.000000       0       0       0       0  0.000000 -2.250901
## 34     Pigr  0.000000       0       0       0       0  0.000000  0.000000
## 35     Mgam  0.000000       0       0       0       0  0.000000  0.000000
## 36   Guca2b  0.000000       0       0       0       0  0.000000  0.000000
## 37   Slc4a5  0.000000       0       0       0       0  0.000000  0.000000
## 38     Ace2  0.000000       0       0       0       0  0.000000  0.000000
## 39  Creb3l3  0.000000       0       0       0       0  0.000000  0.000000
## 40  Tmem236  0.000000       0       0       0       0  0.000000  0.000000
```

```
## 41  Slc5a11  0.000000       0       0       0       0  0.000000  0.000000
## 42   Slc2a2  0.000000       0       0       0       0  0.000000  0.000000
## 43   Npc1l1  0.000000       0       0       0       0  0.000000  0.000000
## 44  Slc15a1  0.000000       0       0       0       0  0.000000  0.000000
## 45  Slc28a1  0.000000       0       0       0       0  0.000000  0.000000
## 46    Mep1b  0.000000       0       0       0       0  0.000000  0.000000
##      YM gWAT   YM mWAT OM iWAT    OM gWAT OM mWAT
## 1   0.000000  0.000000       0   0.000000       0
## 2   0.000000  0.000000       0   0.000000       0
## 3   0.000000  0.000000       0   0.000000       0
## 4   0.000000  0.000000       0   0.000000       0
## 5   0.000000  0.000000       0   0.000000       0
## 6   0.000000  0.000000       0   0.000000       0
## 7   0.000000  0.000000       0   0.000000       0
## 8   0.000000  0.000000       0   0.000000       0
## 9   0.000000  0.000000       0   0.000000       0
## 10  0.000000  0.000000       0   0.000000       0
## 11  0.000000  0.000000       0   0.000000       0
## 12  0.000000  0.000000       0   0.000000       0
## 13  0.000000  0.000000       0   0.000000       0
## 14  0.000000  0.000000       0   0.000000       0
## 15  0.000000  0.000000       0   0.000000       0
## 16  0.000000  0.000000       0  -3.543245       0
## 17  0.000000  0.000000       0 -16.631747       0
## 18  0.000000  0.000000       0  -2.342169       0
## 19  0.000000  0.000000       0  -2.969829       0
## 20  0.000000  0.000000       0  -4.331252       0
## 21  0.000000  0.000000       0  -3.374633       0
## 22  0.000000  0.000000       0   0.000000       0
## 23 -5.554308 -5.801387       0  -5.782714       0
## 24 -2.833874  0.000000       0   0.000000       0
## 25 -2.203462  0.000000       0   0.000000       0
## 26 -2.650428  0.000000       0   0.000000       0
## 27  2.170539  0.000000       0   0.000000       0
## 28  2.290603  0.000000       0   0.000000       0
## 29  0.000000  0.000000       0   0.000000       0
## 30  0.000000  0.000000       0   0.000000       0
## 31  0.000000  0.000000       0   0.000000       0
## 32  0.000000  0.000000       0   0.000000       0
## 33  0.000000  0.000000       0   0.000000       0
## 34  0.000000 -7.272362       0   0.000000       0
## 35  0.000000 -7.670194       0   0.000000       0
## 36  0.000000 -6.750514       0   0.000000       0
## 37  0.000000 -5.571927       0   0.000000       0
## 38  0.000000 -6.530100       0   0.000000       0
## 39  0.000000 -5.876453       0   0.000000       0
## 40  0.000000 -6.829819       0   0.000000       0
## 41  0.000000 -6.647191       0   0.000000       0
## 42  0.000000 -6.371415       0   0.000000       0
## 43  0.000000 -7.391499       0   0.000000       0
## 44  0.000000 -6.874372       0   0.000000       0
## 45  0.000000 -6.570540       0   0.000000       0
## 46  0.000000 -6.326515       0   0.000000       0
```

```r
df_shif2 <- NewList(list_par = list_par_SG_SHIF, list_name = list_name, Genes = Genes)
```

```
##       Genes   OF iWAT OF gWAT OF mWAT  YF iWAT YF gWAT   YF mWAT   YM iWAT
## 1   Eif2s3y -7.718907       0       0 0.000000       0  0.000000  0.000000
## 2     Ddx3y -8.688822       0       0 0.000000       0  0.000000  0.000000
## 3       Uty -5.076700       0       0 0.000000       0  0.000000  0.000000
## 4     Kdm5d -2.281453       0       0 0.000000       0  0.000000  0.000000
## 5       Wt1  0.000000       0       0 7.393220       0  0.000000  0.000000
## 6     Upk3b  0.000000       0       0 7.060477       0  0.000000  0.000000
## 7     Tcf21  0.000000       0       0 4.744298       0  0.000000  0.000000
## 8      Msln  0.000000       0       0 5.999112       0  0.000000  0.000000
## 9     Rspo1  0.000000       0       0 3.500227       0  0.000000  0.000000
## 10     Myrf  0.000000       0       0 3.797087       0  0.000000  0.000000
## 11    Wnt2b  0.000000       0       0 4.587116       0  0.000000  0.000000
## 12    S100g  0.000000       0       0 0.000000       0 -7.928300  0.000000
## 13  Slc10a2  0.000000       0       0 0.000000       0 -5.400816  0.000000
## 14     Car1  0.000000       0       0 0.000000       0 -5.364154  0.000000
## 15     Osr2  0.000000       0       0 0.000000       0 -3.232998  0.000000
## 16     Ggt7  0.000000       0       0 0.000000       0  0.000000  0.000000
## 17   Gm4846  0.000000       0       0 0.000000       0  0.000000  0.000000
## 18     Alpl  0.000000       0       0 0.000000       0  0.000000  0.000000
## 19 Hs3st3a1  0.000000       0       0 0.000000       0  0.000000  0.000000
## 20 Hs3st3b1  0.000000       0       0 0.000000       0  0.000000  0.000000
## 21    Hoxb9  0.000000       0       0 0.000000       0  0.000000  0.000000
## 22    AMPA2  0.000000       0       0 0.000000       0  0.000000  0.000000
## 23   S100a9  0.000000       0       0 0.000000       0  0.000000  0.000000
## 24     Mmp3  0.000000       0       0 0.000000       0  0.000000  0.000000
## 25   Akr1b8  0.000000       0       0 0.000000       0  0.000000  0.000000
## 26      Hdc  0.000000       0       0 0.000000       0  0.000000  0.000000
## 27    Kank4  0.000000       0       0 0.000000       0  0.000000  0.000000
## 28   Fam13a  0.000000       0       0 0.000000       0  0.000000  0.000000
## 29    Muc16  0.000000       0       0 0.000000       0  0.000000 -4.824144
## 30     Gdf3  0.000000       0       0 0.000000       0  0.000000 -4.717367
## 31    Adam8  0.000000       0       0 0.000000       0  0.000000 -3.950195
## 32    Myo1e  0.000000       0       0 0.000000       0  0.000000 -2.701072
## 33    Cadm1  0.000000       0       0 0.000000       0  0.000000 -2.496518
## 34     Pigr  0.000000       0       0 0.000000       0  0.000000  0.000000
## 35     Mgam  0.000000       0       0 0.000000       0  0.000000  0.000000
## 36   Guca2b  0.000000       0       0 0.000000       0  0.000000  0.000000
## 37   Slc4a5  0.000000       0       0 0.000000       0  0.000000  0.000000
## 38     Ace2  0.000000       0       0 0.000000       0  0.000000  0.000000
## 39  Creb3l3  0.000000       0       0 0.000000       0  0.000000  0.000000
## 40  Tmem236  0.000000       0       0 0.000000       0  0.000000  0.000000
## 41  Slc5a11  0.000000       0       0 0.000000       0  0.000000  0.000000
## 42   Slc2a2  0.000000       0       0 0.000000       0  0.000000  0.000000
## 43   Npc1l1  0.000000       0       0 0.000000       0  0.000000  0.000000
## 44  Slc15a1  0.000000       0       0 0.000000       0  0.000000  0.000000
## 45  Slc28a1  0.000000       0       0 0.000000       0  0.000000  0.000000
## 46    Mep1b  0.000000       0       0 0.000000       0  0.000000  0.000000
##      YM gWAT   YM mWAT OM iWAT   OM gWAT OM mWAT
## 1   0.000000  0.000000       0  0.000000       0
## 2   0.000000  0.000000       0  0.000000       0
## 3   0.000000  0.000000       0  0.000000       0
## 4   0.000000  0.000000       0  0.000000       0
```

```
## 5   0.000000  0.000000       0  0.000000       0
## 6   0.000000  0.000000       0  0.000000       0
## 7   0.000000  0.000000       0  0.000000       0
## 8   0.000000  0.000000       0  0.000000       0
## 9   0.000000  0.000000       0  0.000000       0
## 10  0.000000  0.000000       0  0.000000       0
## 11  0.000000  0.000000       0  0.000000       0
## 12  0.000000  0.000000       0  0.000000       0
## 13  0.000000  0.000000       0  0.000000       0
## 14  0.000000  0.000000       0  0.000000       0
## 15  0.000000  0.000000       0  0.000000       0
## 16  0.000000  0.000000       0 -2.980063       0
## 17  0.000000  0.000000       0 -8.393628       0
## 18  0.000000  0.000000       0 -2.153314       0
## 19  0.000000  0.000000       0 -3.456492       0
## 20  0.000000  0.000000       0 -4.442427       0
## 21  0.000000  0.000000       0 -3.148032       0
## 22  0.000000  0.000000       0  0.000000       0
## 23 -5.423431 -6.490555       0  0.000000       0
## 24 -2.557128  0.000000       0  0.000000       0
## 25 -2.534757  0.000000       0  0.000000       0
## 26 -2.057223  0.000000       0  0.000000       0
## 27  2.133064  0.000000       0  0.000000       0
## 28  2.753528  0.000000       0  0.000000       0
## 29  0.000000  0.000000       0  0.000000       0
## 30  0.000000  0.000000       0  0.000000       0
## 31  0.000000  0.000000       0  0.000000       0
## 32  0.000000  0.000000       0  0.000000       0
## 33 -1.770713  0.000000       0  0.000000       0
## 34  0.000000 -7.401248       0  0.000000       0
## 35  0.000000 -8.010751       0  0.000000       0
## 36  0.000000 -7.846187       0  0.000000       0
## 37  0.000000 -7.098005       0  0.000000       0
## 38  0.000000 -7.011510       0  0.000000       0
## 39  0.000000 -6.993476       0  0.000000       0
## 40  0.000000 -6.888190       0  0.000000       0
## 41  0.000000 -6.784540       0  0.000000       0
## 42  0.000000 -6.757304       0  0.000000       0
## 43  0.000000 -6.610091       0  0.000000       0
## 44  0.000000 -6.596487       0  0.000000       0
## 45  0.000000 -6.337082       0  0.000000       0
## 46  0.000000 -6.316311       0  0.000000       0
```

```r
df_shal <- as.data.frame(matrix(0, nrow = nrow(df_shal2), ncol = ncol(df_shal2)))
rownames(df_shal) <- rownames(df_shal2)
df_shal <- as.data.frame(lapply(df_shal2, function(x) ifelse(x != 0, 1, 0)))
colnames(df_shal) <- c("Genes",list_name)
df_shal$Genes <- df_shal2$Genes
```

```r
df_shif <- as.data.frame(matrix(0, nrow = nrow(df_shif2), ncol = ncol(df_shif2)))
rownames(df_shif) <- rownames(df_shif2)
df_shif <- as.data.frame(lapply(df_shif2, function(x) ifelse(x != 0, 1, 0)))
colnames(df_shif) <- c("Genes",list_name)
df_shif$Genes <- df_shif2$Genes
```

```r
df_SHAL_SHIF <- pmax(df_shal,df_shif)
```

```r
df_SHAL_SHIF2 <- df_SHAL_SHIF
rownames(df_SHAL_SHIF2) <- df_SHAL_SHIF$Genes
df_SHAL_SHIF2 <- df_SHAL_SHIF2[, -which(names(df_SHAL_SHIF2) == "Genes")]
```

```r
library(reshape2)
df_SHAL_SHIF_FT <- melt(df_SHAL_SHIF, id.vars = "Genes")
names(df_SHAL_SHIF_FT) <- c("TO", "FROM", "VALUE")
df_SHAL_SHIF_FT <- df_SHAL_SHIF_FT[, c("FROM", "TO", "VALUE")]
df_SHAL_SHIF_FT$VALUE <- as.numeric(df_SHAL_SHIF_FT$VALUE)
```

```r
library(circlize)
library(ggplot2)
library(colorspace)
library(RColorBrewer)
library(ComplexHeatmap)

# Number of pixels based on resolution
dpi <- 300
width <- 800 * dpi / 72
height <- 800 * dpi / 72
png("chord_diagram_SG-SHAL-SHIF.png", width = width,
    height = height, res = dpi,bg = "white")

paleta_colores <- colorRampPalette(brewer.pal(12, "Set3"))
colores <- paleta_colores(length(Genes))
paleta_aleatoria <- sample(colores, length(Genes))
paleta_aleatoria <- c("#D0B28A", "#BCD868", "#E58883", "#CECE66", "#C7949A", "#DAA520",
                      "#EFB36E", "#F2EC84", "#FCFEB3", "#E0DEC5", "#C8A7C9", "#F0EFBB",
                      "#D9EBAE", "#C0BDD8", "#CFBDD0","#E89292", "#8AACCA", "#00CED1",
                      "#C4E8BD", "#A1D3DE", "#A8DDC2", "#F3B962", "#D0EA83", "#A8A0B2",
                      "#CDEBC3", "#C09EBF", "#F0D1E1", "#ACF9A4", "#BBDC76", "#C191C2",
                      "#D0CDCE", "#D9A0AB", "#F0DFD1", "#E6EC99", "#D7A3F7", "#DFD6DB",
                      "#66CDAA", "#E0F3B8", "#FFED6F", "#8DD3C7", "#93B1C1", "#C4B9C1",
                      "#B2B2A5", "#C8D3C3", "#F68378", "#F4A460" )
col_ <- setNames(paleta_aleatoria, Genes)

grid.col = c(c(`OF iWAT` = '#fb6f92', `OF gWAT` = '#f08080', `OF mWAT` = '#ffc2d1',
               `YF iWAT` = '#f77f00', `YF gWAT` = '#fcbf49', `YF mWAT` = '#eae2b7',
               `YM iWAT` = '#335c67', `YM gWAT` = '#0a9396', `YM mWAT` = '#94d2bd',
               `OM iWAT` = '#344e41', `OM gWAT` = '#588157', `OM mWAT` = '#81b29a'), col_)

circos.par(start.degree = 90)
list_sector <- c(rep("OLD FEMALE", 3),rep("YOUNG FEMALE", 3),rep("YOUNG MALE", 3),
                 rep("OLD MALE", 3), rep("Genes", length(Genes)))
sector_names <- c(list_name, Genes)
group <- setNames(list_sector,sector_names)

circos.par(canvas.xlim = c(-0.9, 1.2))
chordDiagram(df_SHAL_SHIF_FT, big.gap = 15, #group = group,
            order = c(rev(colnames(df_SHAL_SHIF2)),
                      rev(rownames(df_SHAL_SHIF2))),
            annotationTrack = "grid",
```

```r
            annotationTrackHeight = 0.04,
            preAllocateTracks = 1, grid.col = grid.col, transparency = 0.3,
            link.lwd = 0.5,     # Line width
             link.lty = 1,     # Line type
             link.border = "#ffffff",
            directional = 1, diffHeight = mm_h(13), #segment height
            target.prop.height = mm_h(5),
            direction.type = c("diffHeight", "arrows"),
            link.arr.type = "big.arrow")


#Rotate label
circos.track(track.index = 1, panel.fun = function(x, y) {
  xlim = get.cell.meta.data("xlim")
  ylim = get.cell.meta.data("ylim")
  sector.name = get.cell.meta.data("sector.index")
  circos.text(mean(xlim), ylim[1] + .14, sector.name, facing = "clockwise",
              niceFacing = TRUE,
              adj = c(0, 0.5), cex = ifelse(sector.name %in% c("OF iWAT", "OF gWAT",
                                                                 "OM iWAT", "OM gWAT",
                                                                 "OM mWAT", "YF gWAT",
                                                                 "YF mWAT", "YM iWAT",
                                                                 "YM gWAT", "YM mWAT",
                                                                 "YF iWAT", "OF mWAT"),
                                              1.1, 0.9))

  circos.axis(h = "bottom",
              labels.cex = 0.01,
              lwd = 0.6,
              labels.pos.adjust = FALSE,
              labels.niceFacing = FALSE)

  if(sector.name %in% "OM gWAT") {
    #sHAL
    circos.rect(ybottom=ylim[1]-2.1, ytop=ylim[2]-2.6, xleft=xlim[1]+0.08,
                xright=xlim[2]-0.05, col = "#0096c7", border = "#0096c7",
                track.index =2)
    #sHIF
    circos.rect(ybottom=ylim[1]-2.7, ytop=ylim[2]-3.19, xleft=xlim[1]+0.08,
                xright=xlim[2]-0.05, col = "#0096c7", border = "#0096c7",
                track.index =2)
    }

  if(sector.name %in% "OM iWAT") {
    #sHAL
    circos.rect(ybottom=ylim[1]-2.1, ytop=ylim[2]-2.6, xleft=xlim[1]+0.08,
                xright=xlim[2]-0.05, col = "#bc4749", border = "#bc4749",
                track.index =2)
    circos.rect(ybottom=ylim[1]-2.1, ytop=ylim[2]-2.6, xleft=xlim[1]+1.03,
                xright=xlim[2]-1.01, col = "#dad7cd", border = "#dad7cd",
                track.index =2)
    #sHIF
    circos.rect(ybottom=ylim[1]-2.7, ytop=ylim[2]-3.19, xleft=xlim[1]+0.08,
```

```
              xright=xlim[2]-0.05, col = "#dad7cd", border = "#dad7cd",
              track.index =2)
  circos.rect(ybottom=ylim[1]-2.7, ytop=ylim[2]-3.19, xleft=xlim[1]+1.03,
              xright=xlim[2]-2.04, col = "#0096c7", border = "#0096c7",
              track.index =2)
  }
if(sector.name %in% "OM mWAT") {
  #sHAL
  #sHIF
  }
if(sector.name %in% "YM mWAT") {
  #sHAL
  circos.rect(ybottom=ylim[1]-2.1, ytop=ylim[2]-2.6, xleft=xlim[1]+0.08,
              xright=xlim[2]-0.05, col = "#0096c7", border = "#0096c7",
              track.index =2)
  #sHIF
  circos.rect(ybottom=ylim[1]-2.7, ytop=ylim[2]-3.19, xleft=xlim[1]+0.08,
              xright=xlim[2]-0.05, col = "#0096c7", border = "#0096c7",
              track.index =2)
  }

if(sector.name %in% "YM gWAT") {
  #sHAL
 circos.rect(ybottom=ylim[1]-2.1, ytop=ylim[2]-2.6, xleft=xlim[1]+0.08,
             xright=xlim[2]-0.05, col = "#0096c7", border = "#0096c7",
             track.index =2)
  circos.rect(ybottom=ylim[1]-2.1, ytop=ylim[2]-2.6, xleft=xlim[1]+6.00,
              xright=xlim[2]-0.05, col = "#dad7cd", border = "#dad7cd",
              track.index =2)
  circos.rect(ybottom=ylim[1]-2.1, ytop=ylim[2]-2.6, xleft=xlim[1]+4.05,
              xright=xlim[2]-1.06, col = "#bc4749", border = "#bc4749",
              track.index =2)
  #sHIF
  circos.rect(ybottom=ylim[1]-2.7, ytop=ylim[2]-3.19, xleft=xlim[1]+0.08,
              xright=xlim[2]-0.05, col = "#0096c7", border = "#0096c7",
              track.index =2)
  circos.rect(ybottom=ylim[1]-2.7, ytop=ylim[2]-3.19, xleft=xlim[1]+4.05,
              xright=xlim[2]-1.06, col = "#bc4749", border = "#bc4749",
              track.index =2)
  }

if(sector.name %in% "YM iWAT") {
  #sHAL
  circos.rect(ybottom=ylim[1]-2.1, ytop=ylim[2]-2.6, xleft=xlim[1]+0.08,
              xright=xlim[2]-0.05, col = "#0096c7", border = "#0096c7",
              track.index =2)
  #sHIF
  circos.rect(ybottom=ylim[1]-2.7, ytop=ylim[2]-3.19, xleft=xlim[1]+0.08,
              xright=xlim[2]-0.05, col = "#0096c7", border = "#0096c7",
              track.index =2)
  circos.rect(ybottom=ylim[1]-2.7, ytop=ylim[2]-3.19, xleft=xlim[1]+0.08,
              xright=xlim[2]-5.05, col = "#dad7cd", border = "#dad7cd",
              track.index =2)
```

```r
  }

  if(sector.name %in% "YF mWAT") {
    #sHAL
    circos.rect(ybottom=ylim[1]-2.1, ytop=ylim[2]-2.6, xleft=xlim[1]+0.08,
                xright=xlim[2]-0.05, col = "#0096c7", border = "#0096c7",
                track.index =2)
    #sHIF
    circos.rect(ybottom=ylim[1]-2.7, ytop=ylim[2]-3.19, xleft=xlim[1]+0.08,
                xright=xlim[2]-0.05, col = "#0096c7", border = "#0096c7",
                track.index =2)
    }

  if(sector.name %in% "YF iWAT") {
    #sHAL
    circos.rect(ybottom=ylim[1]-2.1, ytop=ylim[2]-2.6, xleft=xlim[1]+0.08,
                xright=xlim[2]-0.05, col = "#dad7cd", border = "#dad7cd",
                track.index =2)
    #sHIF
    circos.rect(ybottom=ylim[1]-2.7, ytop=ylim[2]-3.19, xleft=xlim[1]+0.08,
                xright=xlim[2]-0.05, col = "#bc4749", border = "#bc4749",
                track.index =2)
  }

  if(sector.name %in% "YF gWAT") {
    #sHAL
    #sHIF
  }

  if(sector.name %in% "OF gWAT") {
    #sHAL
    #sHIF
    }

  if(sector.name %in% "OF iWAT") {
    #sHAL
    circos.rect(ybottom=ylim[1]-2.1, ytop=ylim[2]-2.6, xleft=xlim[1]+0.08,
                xright=xlim[2]-0.05, col = "#0096c7", border = "#0096c7",
                track.index =2)
    #sHIF
    circos.rect(ybottom=ylim[1]-2.7, ytop=ylim[2]-3.19, xleft=xlim[1]+0.08,
                xright=xlim[2]-0.05, col = "#0096c7", border = "#0096c7",
                track.index =2)
    }

}, bg.border = NA)

#
lgd1 <- Legend(at = c("Depletion", "Enrichment","Not significant"),
        labels_gp = gpar(fontsize = 11),
        legend_gp = gpar(fill = c("#0096c7", "#bc4749","#dad7cd")),
        title_position = "topleft",
        title = expression(bold("SH-AL - SG / SH-IF -  SG (FDR<0.01):")),
```

```
        row_gap = unit(2, "mm"))

draw(lgd1 , x = unit(4, "mm"), y = unit(4, "mm"),
    just = c("left", "bottom"))

lgd2 <- Legend(at = Genes, labels_gp = gpar(fontsize = 11),
    legend_gp = gpar(fill = paleta_aleatoria), title_position = "topleft",
    title = expression(bold("GENES:")), row_gap = unit(1.5, "mm"))

draw(lgd2, x = unit(0.98, "npc"), y = unit(0.97, "npc"),
     just = c("right", "top"))

text(0.1069, 0.628, "SH-AL", pos=4, col="black", cex=0.5, font=2)#, srt=10
text(0.1089, 0.606, "SH-IF", pos=4, col="black", cex=0.5, font=2)#, srt=10
dev.off()
```

```
## pdf
##   2
```

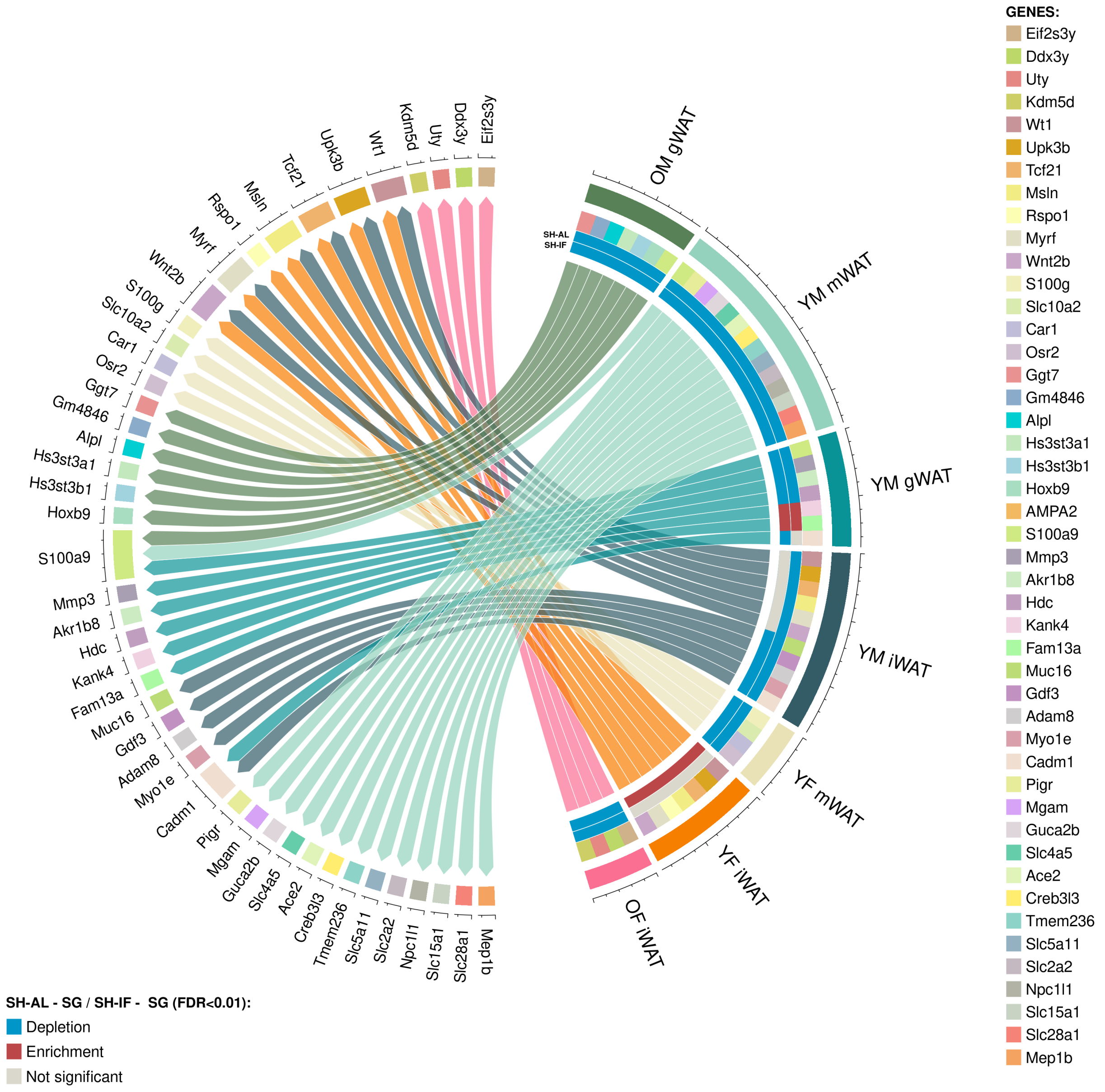
OM gWAT
YM mWAT
YM gWAT
YM iWAT
YF mWAT
YF iWAT
OF iWAT
SH-AL
SH-IF
Eif2s3y
Ddx3y
Uty
Kdm5d
Wt1
Upk3b
Tcf21
Msln
Rspo1
Myrf
Wnt2b
S100g
Slc10a2
Car1
Osr2
Ggt7
Gm4846
Alpl
Hs3st3a1
Hs3st3b1
Hoxb9
S100a9
Mmp3
Akr1b8
Hdc
Kank4
Fam13a
Muc16
Gdf3
Adam8
Myo1e
Cadm1
Pigr
Mgam
Guca2b
Slc4a5
Ace2
Creb3l3
Tmem236
Slc5a11
Slc2a2
Npc1l1
Slc15a1
Slc28a1
Mep1b
GENES:
Eif2s3y
Ddx3y
Uty
Kdm5d
Wt1
Upk3b
Tcf21
Msln
Rspo1
Myrf
Wnt2b
S100g
Slc10a2
Car1
Osr2
Ggt7
Gm4846
Alpl
Hs3st3a1
Hs3st3b1
Hoxb9
AMPA2
S100a9
Mmp3
Akr1b8
Hdc
Kank4
Fam13a
Muc16
Gdf3
Adam8
Myo1e
Cadm1
Pigr
Mgam
Guca2b
Slc4a5
Ace2
Creb3l3
Tmem236
Slc5a11
Slc2a2
Npc1l1
Slc15a1
Slc28a1
Mep1b
SH-AL - SG / SH-IF - SG (FDR<0.01):
Depletion
Enrichment
Not significant
